# Editorial: Recent Advances in Cardiotoxicity Testing

**DOI:** 10.3389/fphar.2021.798189

**Published:** 2021-11-08

**Authors:** Tamer M. A. Mohamed, Javid Moslehi, Jonathan Satin

**Affiliations:** ^1^ Department of Medicine, The Institute of Molecular Cardiology, University of Louisville, Louisville, KY, United States; ^2^ Department of Medicine, Diabetes and Obesity Center, Envirome Institute, University of Louisville, Louisville, KY, United States; ^3^ Department of Cardio-oncology, University of California, San Francisco, San Francisco, CA, United States; ^4^ Department of Physiology, University of Kentucky, Lexington, KY, United States

**Keywords:** cancer, cardiooncology, heart, toxicicity, hiPS cardiomycytes, heart slices

## Introduction

Drug induced cardiotoxicity is a major cause of market withdrawal (Onakpoya et al., 2016). In the last decade of the 20th century, eight non-cardiovascular drugs were withdrawn from clinical use because they prolonged the QT interval (Fermini and Fossa, 2003), resulting in ventricular arrhythmias and potentially sudden death. In particular, the last decade has seen an explosion of cancer therapies, which while effective can lead to several cardiovascular toxicities. Both traditional (e.g., anthracyclines and radiation) and targeted (e.g., trastuzumab) therapies can result in cardiovascular complications in a subset of patients (Groarke et al., 2013; Moslehi, 2016). A close collaboration between cardiologists and oncologists (via the emerging field of “cardio-oncology”) had helped make these complications manageable ensuring that patients can be treated effectively (Campia et al., 2019). An explosion of novel therapies which include newer kinase inhibitors, proteolysis-targeting chimera (PROTAC) and immune-based therapies expand the oncology armamentarium, each drug with its own potential cardiovascular toxicity (Sheng et al., 2016; Fleming et al., 2021). Therefore, there is a growing need for reliable preclinical screening strategies for CV toxicities associated with emerging breast cancer therapies prior to human clinical trials. The most prevalently used cardiac physiology screening platform are animal models that have limited reliability in mirroring the effects of drugs in human hearts (Liu et al., 2006; Salama and Bett, 2014; Asnani et al., 2021). Additionally, the use of animal models to create a pharmacokinetic profile of drugs is relatively expensive at the early development stage since large amounts of the drugs are used (Guth et al., 2019). Ultimately, the ideal experimental cardiac tissue culture model is the one that demonstrates high sensitivity and specificity towards various therapeutic and pharmacological interventions while accurately replicating the physiology and pathophysiology of the human heart (Wang et al., 2008). The recent move towards the use of human induced pluripotent stem cell-derived cardiomyocytes (hiPS-CMs) (Burridge et al., 2016) and human heart slices (Ou et al., 2019; Miller et al., 2020; Ou et al., 2020) in cardiotoxicity testing provided a potential solution to address this issue. In this special issue we have 12 manuscripts, seven review articles and five original research articles summarizing the recent advances in cardiotoxicity testing.

Jaeger et al. used a novel computer simulation to identifying drug response through combining several assessments of the membrane potential, the cytosolic calcium concentration, and the extracellular potential in microphysiological systems. In another effort for in silico modeling of cardiotoxicity, Paci et al., assessed the response of three human stem cell derived cardiomyocytes (hSC-CMs) in silico models to simulate drug action, and compare simulation results against *in vitro* data for 15 drugs. Furthermore, in an effort to create a novel high-throughput platform to detect cardiotoxicity for atrial arrhythmias, Honda et al. (2021) created and validated a high-throughput drug screening system based on hiPS-derived-atrial myocytes. In the same vein, Verkerk et al. optimized a dynamic clamp system to obtain patch-clamp recordings of action potentials from adult primary human atrial myocytes. This technology will be useful in detecting arrhythmogenic cardiotoxins on adult human hearts. The same first author, Dr. Verkerk with other team of collaborators have modeled a prominent cause of arrhythmia in patients with a deficiency in very long-chain acyl-CoA dehydrogenase (VLCAD), an enzyme that is involved in the mitochondrial beta-oxidation of long-chain fatty acids. They generated hiPSC-CMs from VLCAD deficiency patients and they tested the effect of carnitine supplement on attenuating the arrhythmic potential; however, it was not effective.

This special issue includes seven great reviews and perspectives of different topics related to cardiotoxicity testing. Szabo et al. provided a perspective regarding novel anticancer therapeutics to prevent tumorigenesis without cardiotoxic effects highlighting a new family of therapeutics including ERK dimerization inhibitors and BAX allosteric inhibitors. Campana et al. provided a perspective regarding the role of inflammation in cardiotoxicity and how inflammation should be accounted for during drug screening in early stages of drug development. Thomas et al., Keung et al., and Orsolits et al. have provided three comprehensive reviews for the cellular and tissue models derived from hiPS cells and their recent uses to assess cardiotoxicity of cancer therapies. Furthermore, Tu et al. wrote a mini review regarding the use of hiPS-derived cells as a screening platform for drug-induced vascular toxicity. hiPSC-CM systems are ideal for high-throughput testing and for proof-of-principle evaluations of patient-specific genetic backgrounds or disease mutations; however, the incomplete maturation and lack of multicellularity creates caveats. The use of slice preparations is an exciting advance in test platforms because this native tissue preparation retains phenotypic properties *in vitro* (Ou et al., 2019; Miller et al., 2020; Ou et al., 2020). Finally, Meki et al. reviewed the use of adult human and pig heart slices in cardiotoxicity testing.

Overall, this special issue provides an overview of the novel platforms used for cardiotoxicity testing. This field of research is evolving rapidly, and it ultimately holds promise for more reliable and sensitive drug and chemical screening.
